# Improved Expression and Optimization of Trehalose Synthase by Regulation of P_glv_ in *Bacillus subtilis*

**DOI:** 10.1038/s41598-019-43172-z

**Published:** 2019-04-29

**Authors:** Hongling Liu, Hao Liu, Shaojie Yang, Ruiming Wang, Tengfei Wang

**Affiliations:** 1 State Key Laboratory of Biobased Material and Green Papermaking (LBMP), Qilu University of Technology(Shandong Academy of Sciences), Jinan, Shandong, 250353 China; 2 Key Laboratory of Shandong Microbial Engineering, College of Bioengineering, QiLu University of Technology (Shandong Academy of Sciences), Jinan, Shandong, 250353 China; 3 Key Laboratory of Industrial Fermentation Microbiology (Tianjin University of Science &Technology), Ministry of Education, Tianjin, 300457 China

**Keywords:** Metabolic engineering, Bacterial genetics

## Abstract

Trehalose synthase (TreS) converts maltose to trehalose, which has several important functions; therefore, enhancing TreS expression is desirable. Here, a recombinant *Bacillus subtilis* W800N (ΔamyE)-P_glv_ strain was constructed to achieve enhanced expression of TreS. Process optimization strategies were developed to improve the expression level of TreS in *B*. *subtilis* W800N (ΔamyE)-P_glv_. Intracellular activity of TreS was induced using 60 g/L of maltose in shake flask culture. The protein activity reached 5211 ± 134 U/g at 33 °C and pH 7.0 in Luria-Bertani medium. A fed-batch fermentation strategy was applied in a 30 L fermenter containing 18 L terrific broth to achieve high cell density by replacing glycerol with high maltose syrup as a carbon source and an inducer. After 32 h of fermentation, recombinant *B*. *subtilis* W800N (ΔamyE)-P_glv_ activity reached 6850 ± 287 U/g dry cell weight. Our results demonstrate the efficiency of the P_glv_ promoter in increasing the expression of TreS in *B*. *subtilis* W800N (ΔamyE)-P_glv_.

## Introduction

Trehalose is a non-reducing disaccharide comprising two glucose units joined by an α-1,1-glycosidic bond^1^. Functions of trehalose include protection from desiccation^2,3^, stabilization of vaccines^4–6^, and protection of mammalian cells against desiccation^7^. Trehalose is widely used in agricultural, cosmetic, and pharmaceutical industries because of its stability with most chemically nonreactive sugars^8,9^. Trehalose is industrially produced directly from maltose using trehalose synthase (TreS, EC 2.4.1.245) because of the low cost of the substrate and the simplicity of the process, which may be beneficial for industry-scale production^1,10^.

Dual- and single-enzymatic methods are commonly used in the production of trehalose. The drawbacks of the dual-enzymatic method are that it requires the cultivation of two types of recombinant bacteria. These bacteria must be lysed to obtain crude enzymes, which are used for conversion. Thus, the process is associated with the risk of contamination with endotoxins. The single-enzymatic method involves the use of *Escherichia coli* as the expression vector. The removal of endotoxin is also difficult with this method. In addition, isopropyl β-D-1-thiogalactopyranoside (IPTG), the inducer used for the expression of the two enzymes, is expensive and difficult to remove.

TreS is produced by several strains of bacteria, including *Bacillus licheniformis*, *Thermobifida fusca*, and *Yarrowia lipolytica*, which have been considered for use in trehalose production. However, the yield of TreS from bacteria is usually very low, limiting the application of bacterial systems for TreS production. Genetic engineering of bacteria has been carried out with the goal of improving the expression of TreS, including cloning of *treS* gene in *E*. *coli*^11–14^. This approach is time-consuming, and the removal of pathogenic enterobacteria prior to the use of trehalose in the food industry is an expensive process.

*B*. *subtilis* is generally recognized as safe (GRAS) and has been used as a standard host strain because of the ease of cultivation and control over production, which are beneficial factors for large-scale production. *B*. *subtilis* is used in the food industries in many countries because of its GRAS designation. Several inducible expression systems containing inducer-specific promoters, including those for T7, *grac*, *spac*, *xylA*, *sacB*, and *α-amylase* promoter are widely used in *B*. *subtilis*^15–17^. Many proteins have been successfully overexpressed in *B*. *subtilis* using these inducible promoters. However, the high cost of inducer compounds such as IPTG limits their industrial application. The promoter of *glv* operon (P_glv_) in *B*. *subtilis* is positively regulated by maltose, which is inexpensive and widely available. Thus, P_glv_ has been considered as a potential promoter system with industrial applications. However, P_glv_ promoter is markedly repressed by glucose via a catabolism repression element (Cre) located downstream of the transcription origin site of P_glv_ promoter^18–20^.

In this study, the *Cre* gene sequence on P_glv_ promoter was mutated by site-directed mutagenesis^21^. The recombinant plasmid P_glv_-pHT01-treS was constructed as an expression vector. The P_glv_ promoter regulated the expression of TreS in recombinant *B*. *subtilis* W800N (ΔamyE)-P_glv_. Moreover, the conditions for the TreS-catalyzed production of trehalose were optimized.

## Methods

### Bacterial strains, plasmids, primers, and culture media

The bacterial strains, plasmids, and primers used in this study are listed in Table 1. Plasmid pHT01 was purchased from Hangzhou Biosci Biotech Co, Ltd. (Hangzhou, China). *E*. *coli* DH5α and *B*. *subtilis* 168 from our laboratory culture collection were used as hosts for gene cloning. The engineered *B*. *subtilis* WB800N was used for gene expression. Luria-Bertani (LB) medium containing 10 g/L tryptone, 5 g/L yeast extract, and 10 g/L sodium chloride (NaCl) was used as the culture medium. Terrific broth (TB) medium containing 12 g/L tryptone, 24 g/L yeast extract, 4 mL/L glycerol, 2.4 g/L monopotassium phosphate (KH_2_PO_4_), and 16.5 g/L dipotassium phosphate (K_2_HPO_4_) was used as the fermentation medium. Growth medium (GM) containing 10 g/L tryptone, 5 g/L yeast extract, 10 g/L NaCl, and 0.5 mol/L sorbitol was used as the proliferation medium, while regrowth medium RM containing 10 g/L tryptone, 5 g/L yeast extract, 10 g/L NaCl, 0.5 mol/L sorbitol, and 0.38 mol/L mannitol was used as the recovery medium. The electroporation buffer comprised 0.5 mol/L sorbitol, 0.5 mol/L mannitol, 0.5 mol/L trehalose, and 10% glycerol. The antibiotics used for selection were 25 μg/mL chloramphenicol and 50 μg/mL spectinomycin.

**Table 1:** Strains, plasmids, and primers used in this study.

	Relevant properties and Sequence (5′→3ʹ)	Source
**Strains**		
*E*. *coli* DH5α	*deo*R *end*A1 *gyr*A96 *hsd*R17 *sup*E44 *thi*1 *rec*A1 *lac*ZM15	Laboratory collection
*B*. *subtilis* 168	trpC2	Laboratory collection
*B*. *subtilis* W800N	*npr*E *apr*E *epr bpr mpr*:: *ble npr*B:: *bsr* ∆*vpr wpr*A:: *hyg* cm:: neo; Neo^R^	Laboratory collection
**Plasmids**		
pHT01	Plasmid-based expression vector for *B*. *subtilis* containing IPTG-inducible P_grac_ promoter, Cm^R^	Laboratory collection
pHT01-P_glv_-*treS*	P_glv_ promoter instead of P_grac_ promoter	This study
pHT01-P_glv_-*treS* ʹ	Cre sequence (AT base instead of CG base) in P_glv_ promoter	This study
**Primers**		
P_glv_-1-F	CGC*GGATCC*GGCATGTATCCGAATCGT the underlined site is *Bam*HI	This study
P_glv_-1-R-C	CATATGACGACCTCCT*TGATAAATTTTAC*AATTCCATTTATACCATGA	This study
P_glv_-2-F-C	TGTAAAATTTATCAAGGAGGTCGTCATATGAA	This study
P_glv_-2-R-C	TAATAGCGGGCGGAGGGAGCACTTTCACTCCATA AATGAAATTCCCCCATTTTCGAATCTGTAATTGT	This study
TreS-F-C	TTCCCCCATTTTCGAATCTGTAATTGTATATAATAGAAAGAAAATGGGGGGATCTGATATGACCCAGCCCGACCCGT	This study
TreS-R	CCCAAA*GACGTC*TCAAACATGCCCGCTGC the underlined site is *Aat*II	This study
Spe-F	TGGAAACACACAGTGAGGAGGATATATTTGAATACACACGAACA	This study
Spe-R	CGCGGATCCTTATAATTTTTTTAATCCGTTATTTAAATAGTT	This study
amyE-1-F	CGCGGATCCATGTTTGCAAAACGATTCAAAACCT the underlined site is *Bam*HI	This study
amyE-1-R	CAAATATATCCTCCTCACTGTGTGTTTCCATGTGTCCAGTTT	This study

### Construction of the recombinant expression vector pHT01-P_glv_-treS

The *Cre* gene sequence (GTAAACGTTATCA) was embedded in the P_glv_ promoter. In the presence of the *Cre* gene sequence, a glucose metabolism protein (CcpA) inhibits the expression from the P_glv_ promoter. The site-directed mutagenesis of *Cre* gene with CG to AT change^21^ may alleviate the repression of glucose and improve the expression and activity of the protein. Therefore, mutant fragments of P_*glv*_*-*1 (269 bp) and P_*glv*_*-*2 (121 bp) were synthesized by the primer pairs P_*glv*_-1-F/P_*glv*_-1-R-C and P_*glv*_-2-F-C/P_*glv*_-2-R-C using the chromosome of *B*. *subtilis* 168 as the template. A *Bam*HI restriction site was introduced at the 5′-end of fragment P_glv_-1 and the 57 bp homologous fragment upstream of *glvA* gene, and the TAA stop codon and Shine-Dalgarno (SD) sequence of *glv*C gene from *B*. *subtilis* 168 were introduced into the fragment of P_glv_-2. Overlapping polymerase chain reaction (PCR) connected P_glv_-1 and P_glv_-2 fragments to obtain P_glv_-1 + 2 fragments, wherein the overlap section of P_*glv*_-1-R and P_*glv*_-2-F introduced the mutation. The fragment P_*glv*_-1 + 2 created with site-directed mutations in *Cre* sites was obtained through the fusion of P_*glv*_-1 and P_*glv*_-2 overlap PCR fragments using primer pairs P_*glv*_-1-F and P_*glv*_-2-R. The complete fragment comprised a 6-bp enzyme digestion site *Bam*HI sequence added to the corresponding protective bases at the 5′-end of P_*glv*_*-*1 + 2. The *treS* gene from *Pseudomonas putida* ATCC 47054 was amplified by PCR using treS-F/treS-R primer pairs, and the 5′-end of the whole fragment was introduced into *Aat*II digestion site sequence. The P_glv_-*treS* gene fragment was obtained by overlapping PCR with P_*glv*_-1 + 2 and two *treS* gene fragments using P_*glv*_-1 − F and treS-R primer pairs. The resulting 2.5-kb fragment was digested with *Bam*HI and *Aat*II and cloned into pHT01 digested with the same enzymes, resulting in P_*glv*_*-*pHT01-*treS* (Fig. S1).

### Screening of recombinant *B*. *subtilis* WB800N (ΔamyE)

Single-cross integration was used to excise the *amy*E gene from the chromosome of *B*. *subtilis* WB800N. The sequence fragment comprising a 6-bp *Bam*HI digestion site and a 500-bp homologous fragment upstream of *amy*E gene was cloned in *B*. *subtilis* 168 using *amy*E-1-F and *amy*E-1-R primers. The spectinomycin resistance gene was amplified from plasmid pPIC9K using primers Spec-F and Spec-R. These two fragments were fused by overlapping PCR and transformed into the competent *B*. *subtilis* WB800N in LB agar containing 50 μg/mL spectinomycin to screen for spectinomycin-sensitive recombinants. The obtained strain *B*. *subtilis* WB800N (ΔamyE) was analyzed with PCR using primers Spec-F and Spec-R, and subjected to sequencing to confirm the deletion of *amy*E gene (Fig. S2). The integration of *spec* gene at *amy*E locus disrupted the *amy*E gene, resulting in the manifestation of amylase-negative phenotype on LB medium supplemented with 1% starch. After incubation at 37 °C overnight, the plates were stained with iodine to examine amylase activity.

### Preparation of competent recombinant *B*. *subtilis* WB800N

Single colonies of *B*. *subtilis* W800N from LB agar medium were individually inoculated in 5 mL LB liquid medium for 24 h at 37 °C with constant stirring at 200 rpm. A 500-μL aliquot of culture was transferred to 50 mL GM proliferation medium. After the optical density at 600 nm (OD_600_) wavelength reached 1.0, the culture was transferred to a 100-mL centrifuge tube in an ice water bath for 10 min, followed by centrifugation at 4 °C and 5,000 rpm for 8 min to collect bacterial cells. The bacteria were washed thrice with pre-cooled ETM electroporation buffer and finally suspended in 500 μL of ETM buffer to obtain competent *B*. *subtilis*. The competent bacteria were stored at −80 °C (60 μL per tube).

### Construction of recombinant strains

One tube each of 60 μL of *B*. *subtilis* WB800N and *B*. *subtilis* WB800n (ΔamyE) was recovered from −80 °C freezer and uniformly mixed with 6 μL of pHT01-P_glv_-*treS* and pHT01-P_glv_-*treS* plasmid solution. After pre-cooling for 5 min, the mixed liquid was added to a 2-mm electroporation cup. The samples were subjected to electric shock at 1,500 V and 5 ms with an Eppendorf electric rotating apparatus. After electroporation, the electroporation mixture was rapidly mixed with 1 mL of RM medium at 37 °C and 180 rpm for 4 h. The recombinant bacteria were recovered by centrifugation and grown on LB agar medium containing chloramphenicol (25 μg/mL) at 37 °C for 48 h. Chloramphenicol-resistant bacteria were recovered and the recombinant plasmid in the bacteria was extracted for use as template. The *treS* gene was amplified by *treS*-F-C/*treS*-R primer to obtain a positive clone of *B*. *subtilis* WB800N.

### Induced expression and optimization of shake flask induction conditions

Each recombinant plasmid was transformed into *B*. *subtilis* W800N and *B*. *subtilis* W800N (ΔamyE), yielding different TreS-producing strains. A single colony of different recombinant strains selected using LB agar containing antibiotics was inoculated into a 250-mL shaking flask containing 50 mL LB liquid medium with chloramphenicol. The primary seed culture was obtained by incubation at 37 °C for 12 h. A total of 1 mL of primary seed culture of each recombinant strain was transferred to a 500-mL shake flask containing 100 mL LB medium supplemented with 25 μg/mL chloramphenicol and cultured at 37 °C overnight on a rotating oscillator (200 rpm/min) until the OD_600_ value reached 1.2. Maltose was added at a final concentration of 60 g/L to induce protein expression during the subsequent 18 h cultivation at 27 °C, 30 °C, 33 °C, and 37 °C to examine the influence of temperature on protein expression. In another experiment, final maltose concentrations of 20–100 g/L were used to induce the expression of target protein at 37 °C. Maltose was added (final concentration of 60 g/L) at various time points during the cultivation of the recombinant strains at 33 °C to examine the influence of time of maltose addition on growth and TreS production. Samples were collected at certain intervals, and OD_600_ and enzyme activities were measured. Each reported value represents an average of two or three independent measurements and does not vary from the mean value by over 10%.

### Fed-batch fermentation

The recombinant *B*. *subtilis* W800N (ΔamyE)-P_glv_ strain was used to scale-up fermentation in 30 L fermenters (Bailun Biological Technology Co. Ltd., China) containing 20 L of fermentation medium and 100 μg/mL of chloramphenicol. Seed culture in LB medium was inoculated (5% v/v) into TB medium for cultivation. The dissolved oxygen concentration was maintained between 10% and 30% by stirring (0–650 rpm) and a constant sterile air flow rate (0.2–1 vessel volume/min) maintained. A pH value of 7.0 + 0.2 was maintained using automatic pH control with the simultaneous addition of 250 g/L sodium hydroxide solution and 25% hydrochloric acid solution. Temperature was controlled at 37 °C for cell growth and expression. TreS was expressed using a heating tube and integrated cooling system from 37 °C to 33 °C. High concentration of maltose syrup containing 88–90% maltose, 2.5–4% glucose, and 4–6% polysaccharide was used as inducer because of its low cost and easy availability. Samples were obtained every 2 h to analyze the dry cell weight and TreS enzyme activity after induction. After fermentation, the culture was centrifuged (8,000 rpm for 20 min), washed twice with distilled water, and dried to a constant weight at 80 °C to determine the biomass of the culture in terms of dry cell weight.

### Sodium dodecyl sulfate polyacrylamide gel electrophoresis (SDS-PAGE) and TreS activity

The cultured bacteria were harvested by centrifugation at 8,000 rpm for 20 min at 4 °C. The OD_600_ of every sample was measured, and a volume corresponding to approximately the same OD_600_ was harvested and centrifuged. The pelleted cells were resuspended in phosphate-buffered saline (PBS, pH 8.0) and disrupted by sonication on ice using 5 s pulses at an interval of 5 s for 15 min. The crude cell extract was separated by centrifugation and subjected to SDS-PAGE according to the standard procedure. The amount of TreS produced was determined by staining the gels with Coomassie Brilliant Blue R250.

### Preparation, separation, and crystallization of TreS

To identify the enzymatic characteristics of TreS, the enzyme was purified by nickel-nitrilotriacetic affinity chromatography for 1 h using maltose (100 g/L final concentration) as substrate at different pH and temperature values. The enzyme activity of TreS at different temperatures was studied in 100 mM phosphate buffer (pH 8.0) during a 60-min incubation using 100 g/L maltose as substrate. To examine the thermal stability of TreS, TreS was pre-incubated at temperatures from 10 °C to 60 °C for 60 min at pH 8.0. Residual TreS activity was measured at 25 °C. The optimum temperature for enzyme activity was 25 °C, and the enzyme activity was relatively stable from 10 °C to 40 °C. However, TreS activity rapidly decreased with time as the reaction temperature exceeded 40 °C (Fig. S3). The enzyme activity of TreS at various pH values was studied at a constant temperature of 25 °C in 100 mM phosphate buffer (pH 3.0–10.0) for 60 min using 100 g/L maltose as substrate. To examine the temperature and pH stability of TreS, the enzyme was pre-incubated in buffers with pH varying from 3.0 to 10.0 for 60 min at 25 °C. Residual TreS activity was measured at pH 8.0. The optimal pH for enzyme activity was 8.0, and the enzyme activity was relatively stable between pH 6.5 and 9.0 (Fig. S4).

To obtain trehalose at high purity, the pH of the conversion system containing the mixed syrup was adjusted to 5.0 with dilute hydrochloric acid, and the enzyme was denatured by heating at 80 °C for 10 min. The polymer particles were filtered to clarify the mixed sugar liquid. The residual maltose in the mixed sugar solution was hydrolyzed to glucose by glucoamylase (200 U/g maltose) at 62 °C ± 1.0 °C and pH 4.5 and reacted for 6 h to obtain a mixed sugar solution comprising glucose, trehalose, a small amount of maltose, and polysaccharide. At a temperature of 75 °C ± 1.0 °C, 0.15 g of activated carbon was adsorbed per 100 mL for 60 min and the residual metal ions were removed with a cation-exchange resin (001 × 7, Purilit Co., Ltd.) and an anion-exchange resin (D301, Purilit Co., Ltd.) positioned in series. The mixed sugar solution was first passed through a cationic resin and then neutralized with H^+^ and OH^−^ ions using anionic resin. In the subsequent step, a 0.5 nm NF2 nanofiltration membrane system (Beijing Ande Membrane Separation Technology and Engineering Co., Ltd.) was used to separate glucose, trehalose, and the small amount of polysaccharide at 30 °C to obtain a highly pure trehalose solution. The trehalose syrup was concentrated to 80% (w/w) with a plate evaporator and the crystals were slowly cooled. As the temperature of trehalose syrup fell to 60 °C, the trehalose syrup was allowed to cool to 30 °C at a rate of 0.5 °C/h. The crystalline trehalose was obtained by centrifugation and rapidly washed with a small volume of ice water. The collected samples were dehydrated at temperature from 55 °C to 60 °C in a fluidized bed reactor. The maltose in the mixed syrup was hydrolyzed to glucose with glucoamylase (200 U/g maltose) and filtered through a flat filter at 80 °C, followed by concentration to 80% (v/v) by evaporation in vacuo.

### High-performance liquid chromatography (HPLC) analysis of trehalose production

The maltose solution (300 g/L) was treated with crude enzyme (5 mL) at 25 °C for 1 h, followed by incubation in boiling water for 15 min. The solution was centrifuged at 13,000 rpm for 15 min, and the supernatant was analyzed by HPLC, as previously described^22^. The transition rate of trehalose was calculated as follows:

$${\rm{\alpha }}=\frac{{m}_{trehalose}}{{m}_{trehalose}+{m}_{glucose}\frac{342}{360}+{m}_{maltose}}\times 100 \% $$

where *m*_*trehalose*_, *m*_*glucose*_, and *m*_*maltose*_ are weights of trehalose, glucose, and maltose, respectively.

## Results

### Expression of TreS

**Figure 1:**
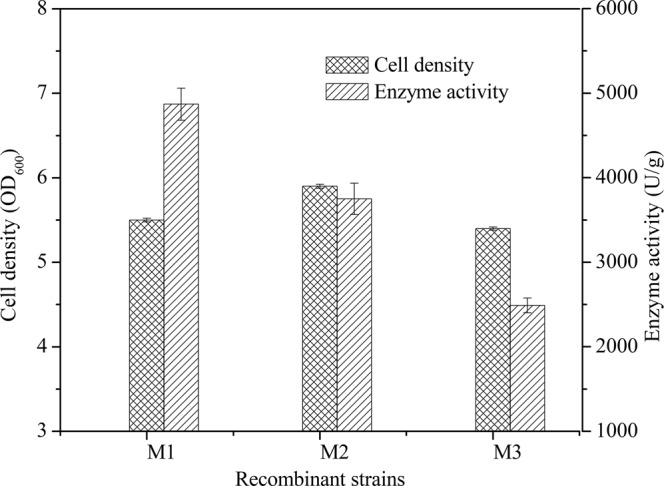
Comparison of enzyme activities in different strains of recombinant *B*. *subtilis* (n = 3, x ± SD).

**Figure 2:**
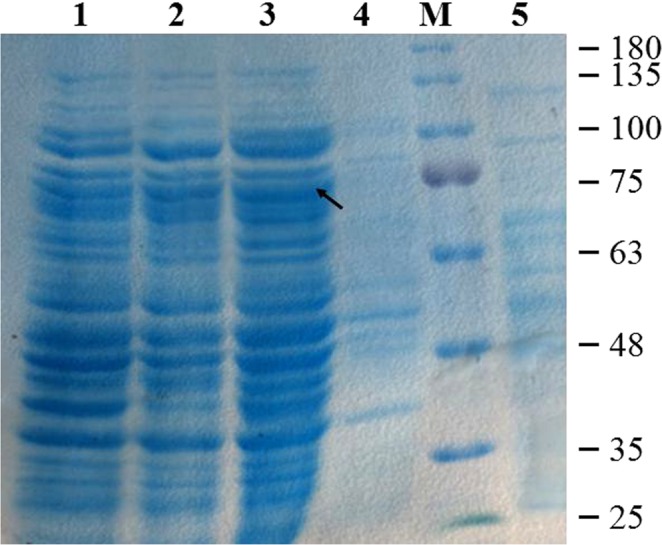
SDS-PAGE analysis of TreS from different recombinant strains. Lane M, protein molecular mass marker; Lane 1, intracellular proteins of recombinant *B*. *subtilis* W800N-P_grac_; Lane 2, intracellular proteins of recombinant *B*. *subtilis* W800N-P_glv_; Lane 3, intracellular proteins of recombinant *B*. *subtilis* W800N (ΔamyE)-P_glv_; Lane 4. proteins in supernatant of recombinant *B*. *subtilis* W800N-P_glv_ fermentation broth; and Lane 5, proteins in supernatant of recombinant *B*. *subtilis* W800N (ΔamyE)-P_glv_ fermentation broth. TreS is indicated by an arrow. Molecular mass markers indicated from top to bottom.

The recombinant clones (*B*. *subtilis* W800N (ΔamyE)-P_glv_, M1; *B*. *subtilis* W800N-P_glv_, M2; and *B*. *subtilis* W800N (ΔamyE)-P_grac_, M3) were subjected to fermentation followed by sonication to extract intracellular proteins. Comparison of enzyme activity of the recombinant *B*. *subtilis* strains (Fig. 1) showed that the enzyme activity of M1 reached 4,870 ± 189 U/g dry weight with 60 g/L maltose as the induction substrate after 18 h, higher than that of M2 (3750 ± 185 U/g) using 60 g/L maltose as the induction substrate and M3 (2490 ± 87 U/g) in the presence of 0.5 mM IPTG as inducer. The expression of recombinant TreS was confirmed by 10% SDS-PAGE. The recombinant proteins had a molecular weight of nearly 75 kDa (Fig. 2), consistent with the molecular weight deduced from the amino acid sequence of TreS. These data suggest that the P_glv_ promoter was superior to P_grac_ promoter in enhancing TreS expression and that the knockout of *amyE* gene further increased the expression of TreS in the recombinant group. As the highest yield was obtained from *B*. *subtilis* W800N (ΔamyE)-P_glv_, the construct (ΔamyE)/pHT01-P_glv_-treS was chosen for further studies.

### Optimization of conditions for recombinant enzyme production

To identify the optimal conditions for recombinant enzyme production, pH, induction temperature, maltose induction concentration, and time of induction were assessed.

**Figure 3:**
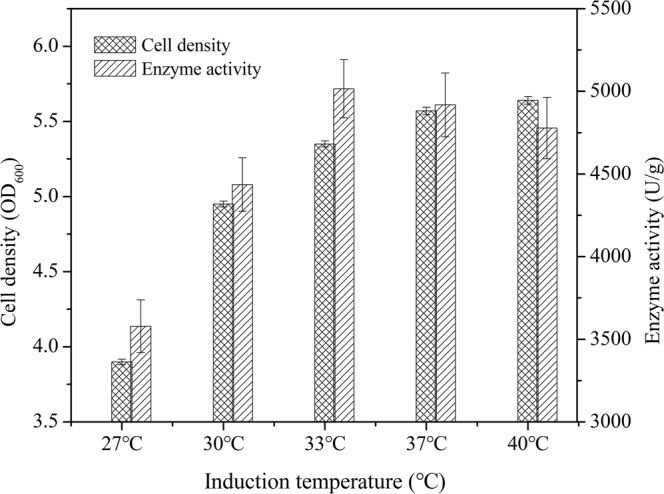
Effect of temperature on the intracellular activity and growth of cells (n = 3, x ± SD).

Temperature is an important parameter for recombinant protein expression in host bacteria. In general, the growth of host bacteria at low temperatures lowers the rate of synthesis of recombinant proteins and increases exogenous protein solubility. To determine the optimal temperature for TreS production, M1 was induced at 27 °C, 30 °C, 33 °C, 37 °C, and 40 °C in LB medium. The highest cell density (OD_600_) of 5.6 ± 0.23 was obtained at 40 °C, while the maximum activity of 4918 ± 193 U/g was reported at 33 °C (Fig. 3). Although temperatures of 37 °C and 40 °C may be more suitable for the growth of bacteria, the lower enzyme activity per gram weight limits the total enzyme activity. Therefore, M1 was more conducive for expression at a temperature of 33 °C.

**Figure 4:**
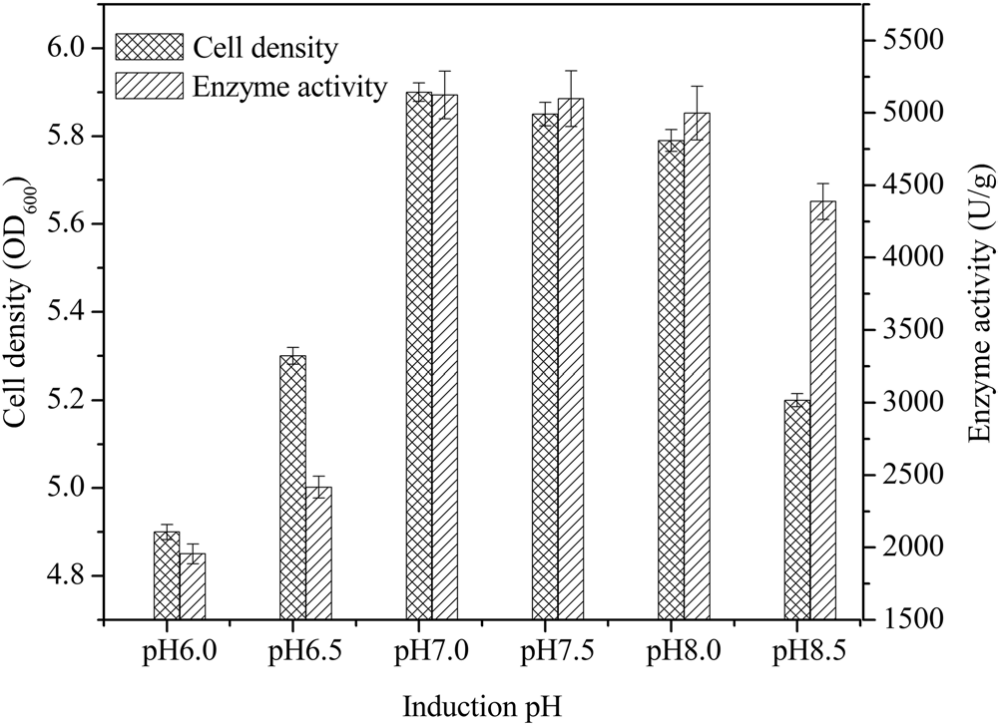
Effect of pH on the intracellular activity and growth of cells (n = 3, x ± SD).

The pH adaptability of M1 was investigated using a standard assay. The enzyme activity was maximum at pH 7.0 and rapidly increased between pH 6.5 and 7.0 (Fig. 4); no significant changes were reported between pH 7.0 and 8.0. In this expression system, both weak acidic conditions (pH < 6.5) and alkaline conditions (pH > 8.8) strongly inhibited the activity of TreS.

**Figure 5:**
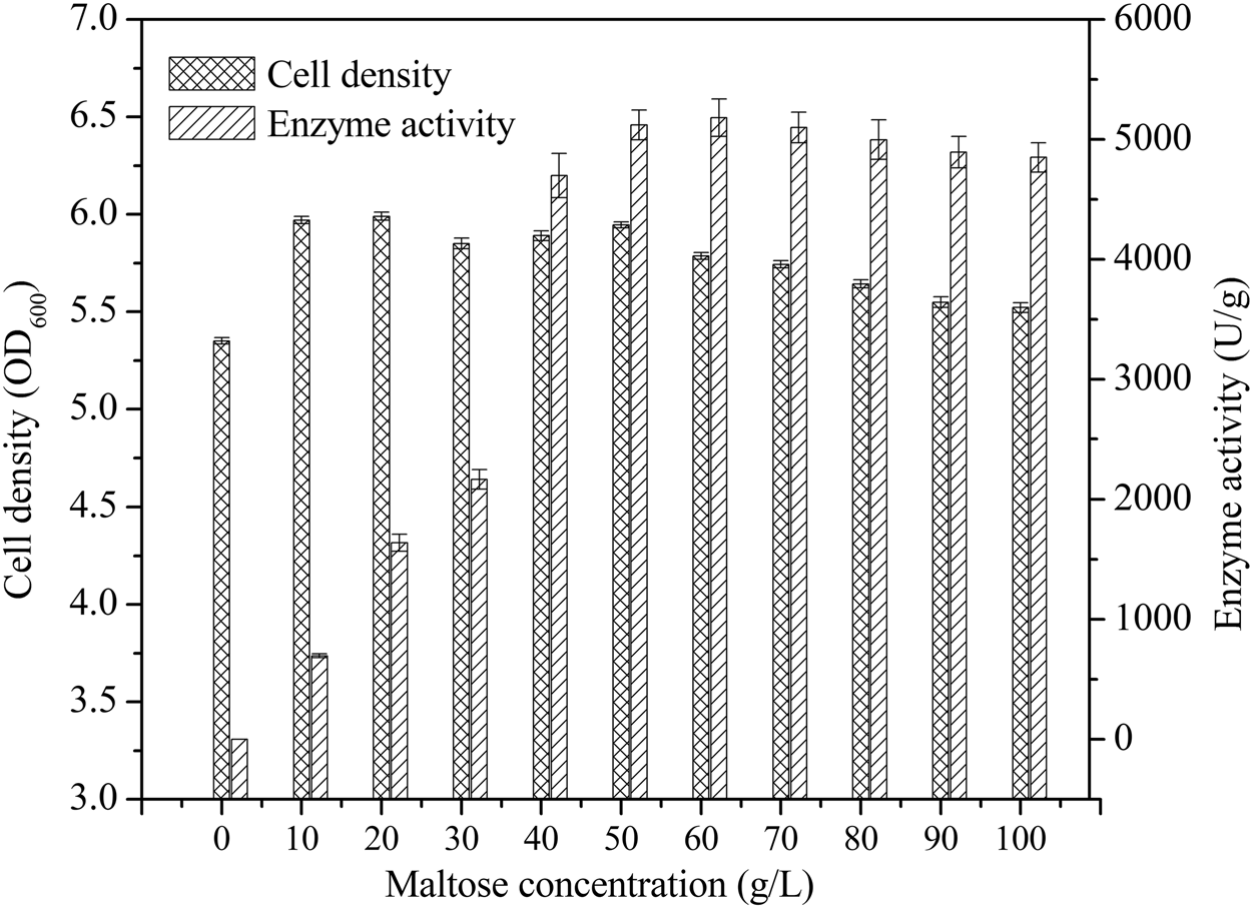
Effect of different maltose concentrations on the enzyme activity of recombinant *B*. *subtilis* W800N (ΔamyE)-P_glv_ (n = 3, x ± SD).

The expression of TreS was induced by maltose; different concentrations of maltose were used to identify the optimum concentration of inducer. TreS activity increased with an increase in maltose concentration and an optimum activity of 5189 ± 197 U/g was achieved following protein expression induction using 60 g/L maltose (Fig. 5). However, further increase in maltose concentration reduced the activity of TreS. In contrast, maltose concentration influenced bacterial growth, but no significant changes were evident at the maltose concentrations used in the present study.

**Figure 6:**
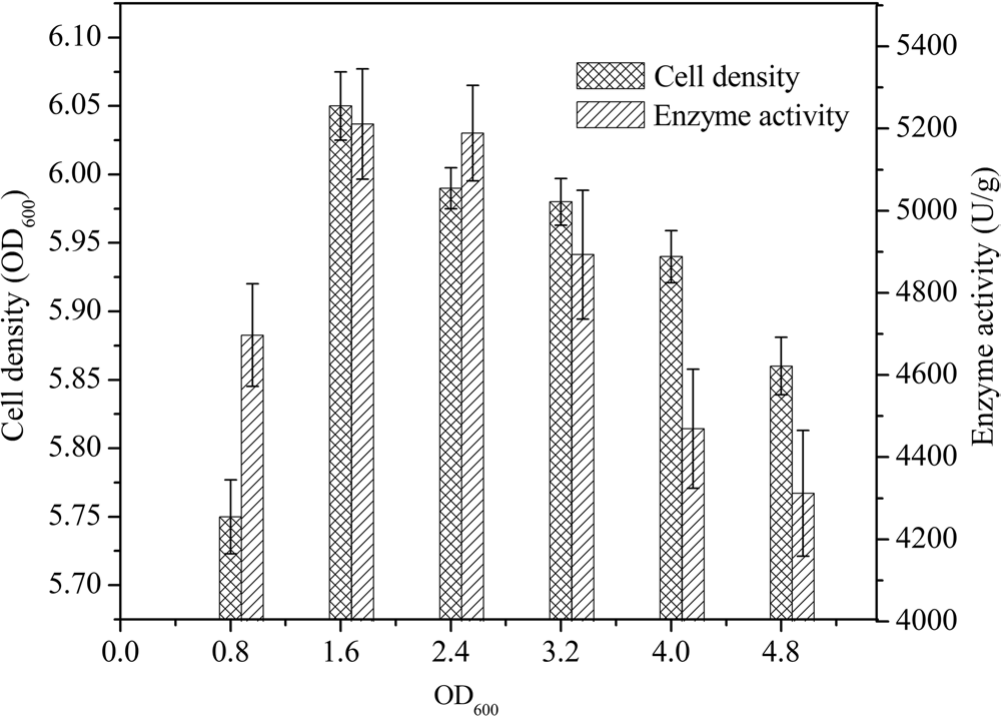
Effect of inducer addition time on the intracellular activity and growth of cells (n = 3, x ± SD).

A set of induction experiments was performed using M1 to determine the optimum dose of the inducer xylose. All induced samples were analyzed for TreS activity and cell density after fermentation. As shown in Fig. 6, the activity was high upon addition of xylose to the culture in the mid-logarithmic phase of growth. The maximum activity of 5211 ± 134 U/g was obtained by adding xylose at an OD_600_ of 1.6.

### Scale-up production of TreS

**Figure 7:**
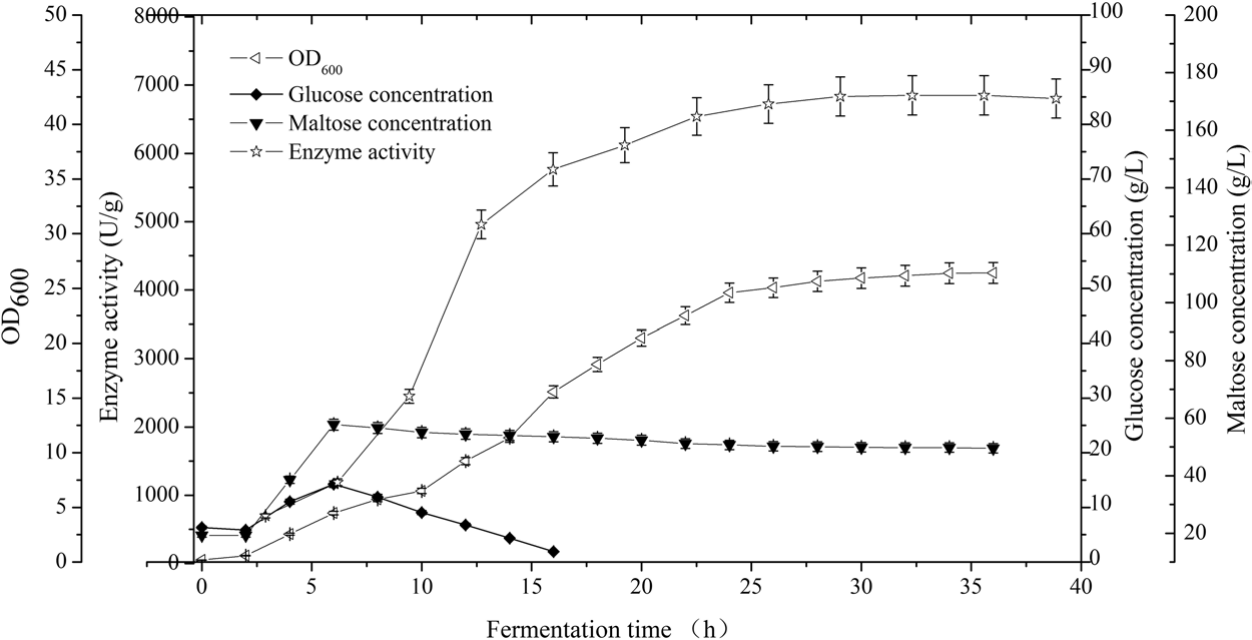
Cell growth and intracellular activity of TreS produced in batch mode, followed by fed-batch cultivation (n = 3, x ± SD).

To scale-up the production of TreS, M1 culture was induced to express TreS in a 20 L fermenter containing glycerol-free TB medium (Fig. 7). High maltose concentration was added to the medium in three phases. The first phase was started in the presence of an initial high maltose syrup concentration of 10 g/L. After the OD_600_ reached approximately 1.6, the second dose of high maltose syrup was added at a concentration not exceeding 40 g/L. After 6 h of fermentation, the third dose of high maltose syrup was added at a concentration of 64 g/L. In the second phase, the feed medium (TB medium without glycerol) was used until the OD_600_ value reached 25 and the temperature decreased to 33 °C. Intracellular TreS enzyme activity reached 6850 ± 287 U/g after 32 h fermentation, a value that was 1.3 times the level attained in shake flask cultivation.

### Purification of trehalose

**Figure 8:**
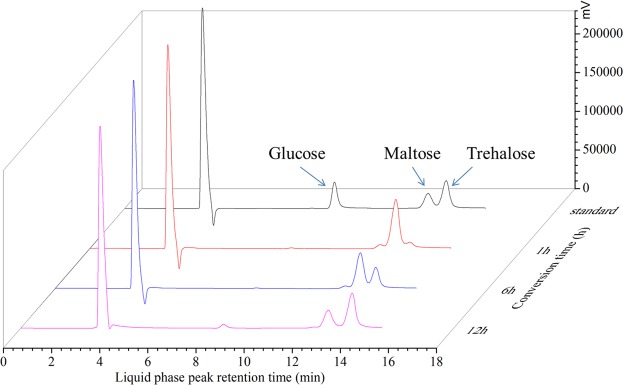
HPLC analysis of mixed syrup in the process of conversion.

**Figure 9:**
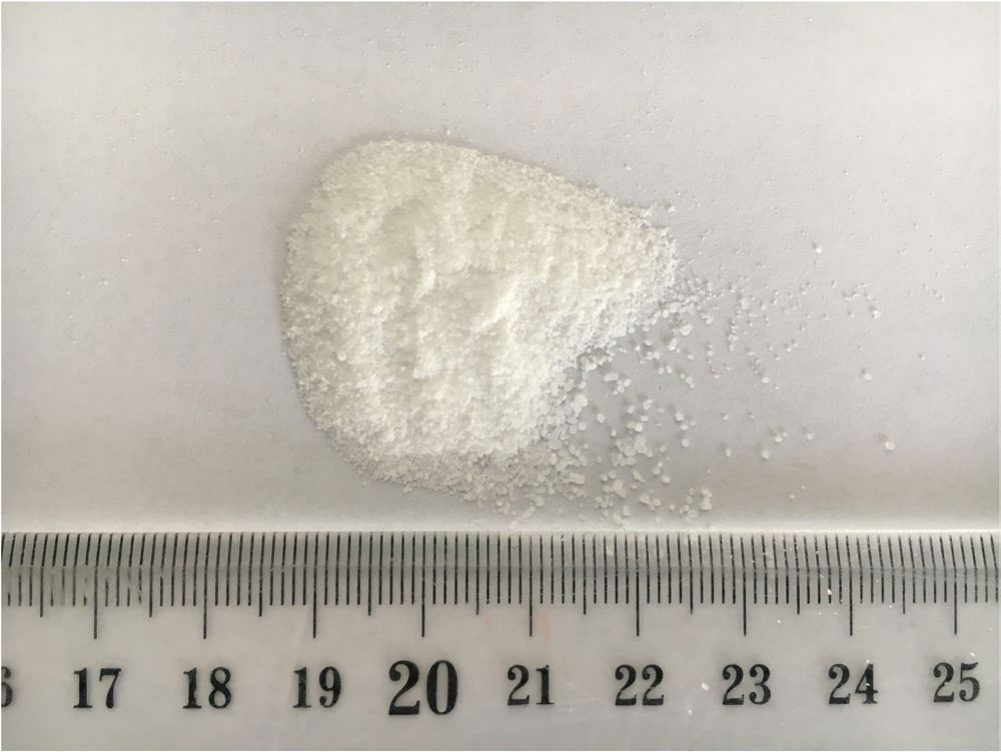
Trehalose product analysis. (**A**). Trehalose product crystallization. (**B**). Analysis of HPLC chromatogram from trehalose purification.

As the optimum temperature for TreS expression was determined to be 25 °C, the enzyme exhibited competent transformation activity at low temperatures. To avoid the growth of microorganisms at room temperature (25 °C) or to prevent any effect on transformation, 15 °C was chosen as the reaction temperature. Recombinant *B*. *subtilis* W800N cell lysate containing TreS was directly used to convert high maltose syrup for 12 h at 15 °C and pH 8.0 with agitation at 60 rpm in the presence of 300 U TreS/g maltose and 300 g/L high maltose syrup. After conversion in the mixed tank for 12 h under optimum conditions, a mixture of glucose, maltose, and trehalose was obtained. The maximal conversion efficiency of TreS was 67% (Fig. 8). After crystallization, the purified trehalose (≥ 99%) was analyzed by HPLC (Fig. 9).

## Discussion

Endospore-forming bacteria of the genus *Bacillus* are used to manufacture several industrial enzymes, in particular amylase and protease. In the majority of cases, a constitutive expression pattern that couples growth and expression is employed^23^. Plasmid pHT01 is a versatile *E*. *coli-B*. *subtilis* shuttle vector. The full-length of 7,955 bp is produced using P_grac_ as the control element and IPTG as the inducer. Although most published studies have focused on the use of IPTG as the inducer^11,24,25^, IPTG is a potentially toxic chemical and is expensive. To avoid these drawbacks, we constructed a new recombinant expression vector, pHT01-P_glv_, using P_glv_ as the control element instead of P_grac_. P_glv_ was fused to a synthetic ribosome-binding site fragment and tandem stop codons were introduced to efficiently avoid read-through transcription. The presence of unique restriction sites downstream of the maltose-inducible promoter allows the proper ligation of coding regions for target protein overproduction. *B*. *subtilis* WB800N can secrete α-amylase to hydrolyze maltose and form glucose in the medium, resulting in the marked reduction in the induction efficiency of maltose^20^. Therefore, it is necessary to knockout *amyE* gene in the chromosome of *B*. *subtilis* WB800N. The spectinomycin gene was integrated into the chromosome of *B*. *subtilis* WB800N at *amyE* locus by a single crossover event. Amylase activity analysis confirmed the interruption of the complete expression of amylase, resulting in the abrogation of amylase activity. The recombinant *B*. *subtilis* strains W800N-P_glv_ and W800N-P_grac_ described in the present study produced functional TreS in the presence of simple fermentation media containing xylose as inducer. Enzyme activity was the highest for the strain *B*. *subtilis* W800N (ΔamyE)-P_glv_, suggesting that the P_glv_ promoter is more efficient in expression the of TreS than the P_grac_ promoter.

Temperature and pH are usually the main factors that determine the production efficiency of TreS in recombinant hosts^26^. The present findings are consistent with those previously reported on the optimum temperature of approximately 33 °C for TreS expression in different hosts. At present, TreS cloned from *B*. *subtilis* W800N (ΔamyE)-P_glv_ showed the highest activity at pH 7.0, similar to the activities observed for the enzymes from *Mycobacterium smegmatis* (pH 7.0)^27^ and *Corynebacterium glutamicum* ATCC 13032 (pH 7.0)^14^.

In general, fed-batch culture is used in bio-industrial processes to achieve high cell density in bioreactors^26^. The fermentation period of recombinant *E*. *coli* BL(DE3) was only 14 h and the time to add the inducer of protein expression was easy to calculate. Lactose, as an inducer instead of IPTG, is more economical and easier to obtain^28^. Furthermore, lactose can be completely consumed as a carbon source during the fermentation process, leaving no residue or product in the medium^29^. The recombinant *B*. *subtilis* W800N (ΔamyE)-P_glv_ described herein produced functional TreS on a simple fermentation medium containing xylose as the inducer. The maximum TreS activity was obtained upon the addition of xylose at an OD_600_ value of 1.6.

Crude TreS solutions were directly used for conversion, thereby avoiding the process of purification of TreS. TreS was purified by nickel-nitrilotriacetic affinity chromatography for 1 h using maltose a substrate at different pH and temperature values. The pH of the conversion system containing mixed syrup was adjusted to 5.0, resulting in the production of very pure trehalose. The trehalose solution was concentrated by a plate-type evaporator and cooled to crystallize the product. The purity of the produced trehalose exceeded 99%.

## Conclusions

We obtained a recombinant *B*. *subtilis* W800N (ΔamyE)-P_glv_ for the intracellular expression of TreS using P_glv_ as the promoter and maltose as the inducer. The recombinant *B*. *subtilis* W800N (ΔamyE)-P_glv_ efficiently expressed TreS, indicating that the mutant P_glv_ can evidently alleviate the repression caused by glucose to enhance the expression of the P_glv_ promoter system. In the food and pharmaceutical industries, processes that are inexpensive, simple, and free of endotoxins are key. The direct use of recombinant *B*. *subtilis* W800N cell lysates to prepare trehalose from maltose takes into account these criteria. Thus, the expression of TreS by recombinant *B*. *subtilis* W800N using maltose as an inducer not only provides a safe and efficient expression system for TreS but also provides a new direction for the industrial production of trehalose.

## Supplementary information

Supplementary Information files

## Data Availability

All data generated or analyzed during this study are included in this published article (and its Supplementary Information files).
