# Supplementary material for: Improved Expression and Optimization of Trehalose Synthase by Regulation of Pglv in Bacillus subtilis
Source: Sci Rep. 2019 Apr 29;9:6585. doi: 10.1038/s41598-019-43172-z (PMC6488592; doi:10.1038/s41598-019-43172-z)

**Improved Expression and Optimization of Trehalose Synthase by Regulation of P_glv_ in *Bacillus subtilis***

**Running Title: P_glv_ regulates trehalose production in *B. subtilis***

Hongling Liu^1,2^ , Hao Liu^1,2^, Shaojie Yang^3^, Ruiming Wang^3^, Tengfei Wang^3*^

^1^Key Laboratory of Industrial Fermentation Microbiology (Tianjin University of Science & Technology), Ministry of Education, Tianjin, 300457, China

^2^Key Lab of Industrial Microbiology, Tianjin University of Science and Technology, Tianjin, 300457, China

^3^Key Laboratory of Shandong Microbial Engineering, QILU University of Technology, Jinan, Shandong, 250353, China

^*^ **Corresponding author**

Tengfei Wang

Key Laboratory of Shandong Microbial Engineering, QILU University of Technology, No. 3501, University Road, Changqing District, Jinan, Shandong, 250353, P.R. China

Tel: +86-531-89631076

Fax: +86-531-89631076

Email: wangtengfei1981@163.com

**Improved Expression and Optimization of Trehalose Synthase by Regulation of P_glv_ in *Bacillus subtilis***

**Running Title: P_glv_ regulates trehalose production in *B. subtilis***


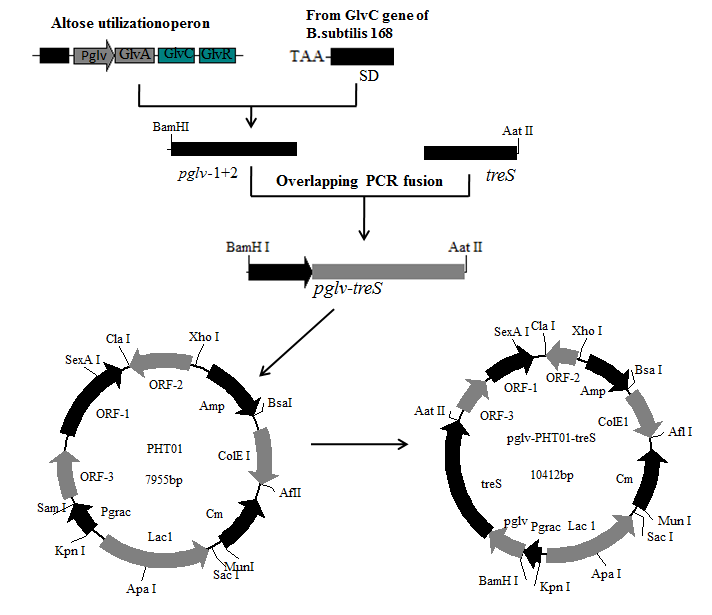


Supplementary Figure 1 The construction sketch map of recombinant plasmid pHT01-P_glv_-*treS* for the expression of TreS in *B. subtilis* W800N


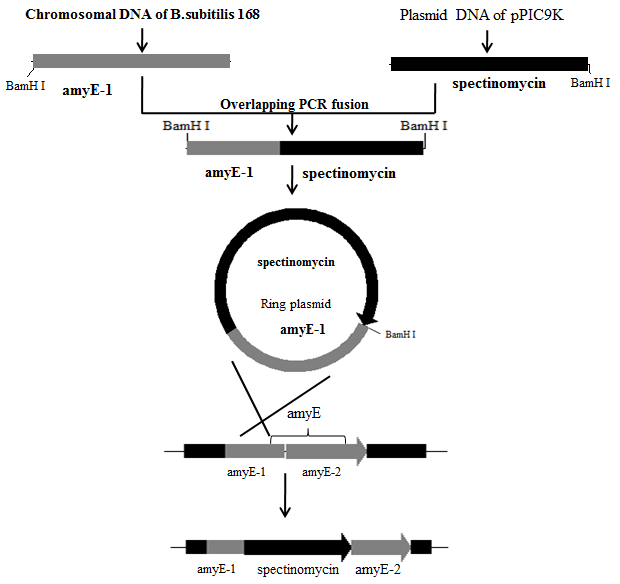


Supplementary Figure 2. Schematic diagram of *spec* gene fusion integrated at *amy*E locus in the *B. subtilis* chromosome.


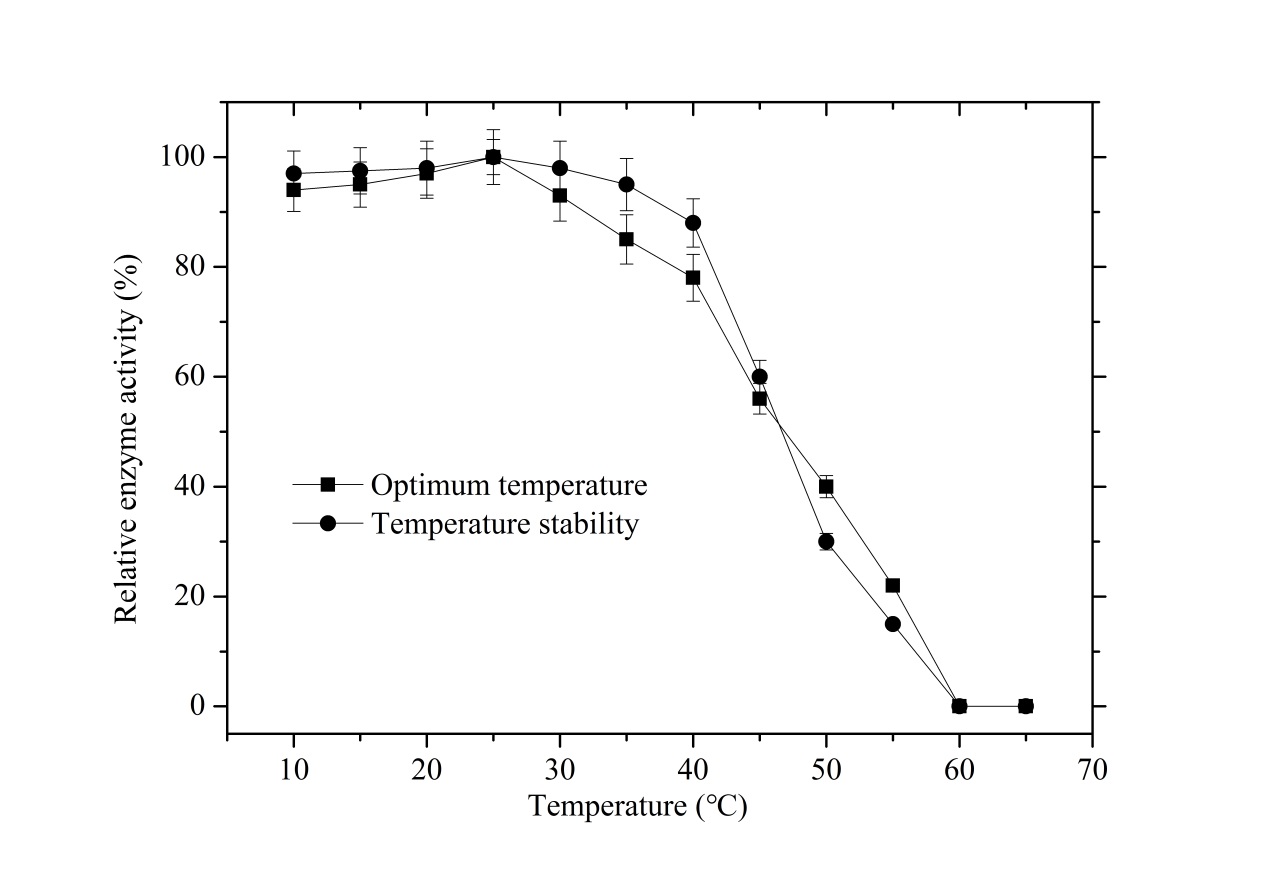


Supplementary Figure 3 Effect of temperature on the relative activity and temperature stability of TreS. Relative activity was calculated by assuming the activity obtained at 25°C. The average of relative values is shown (n = 3, x ± SD).


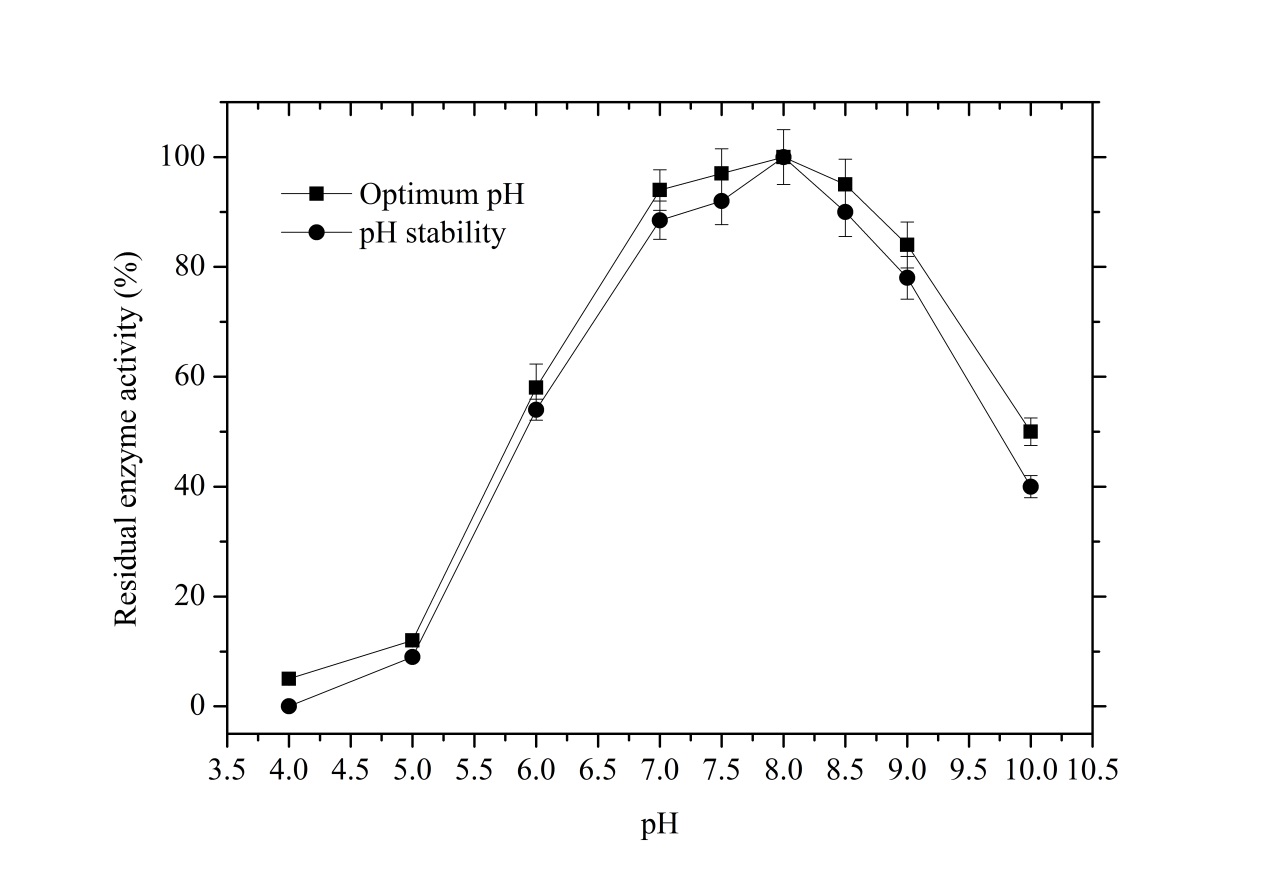


Supplementary Figure 4 Effect of pH on the relative activity and pH stability of TreS. Relative activity was calculated by assuming the activity obtained at pH 8.0 as 100%. The average of relative values is shown (n = 3, x ± SD)

**Supplementary materials below are typical full blot of the SDS-PAGE:**


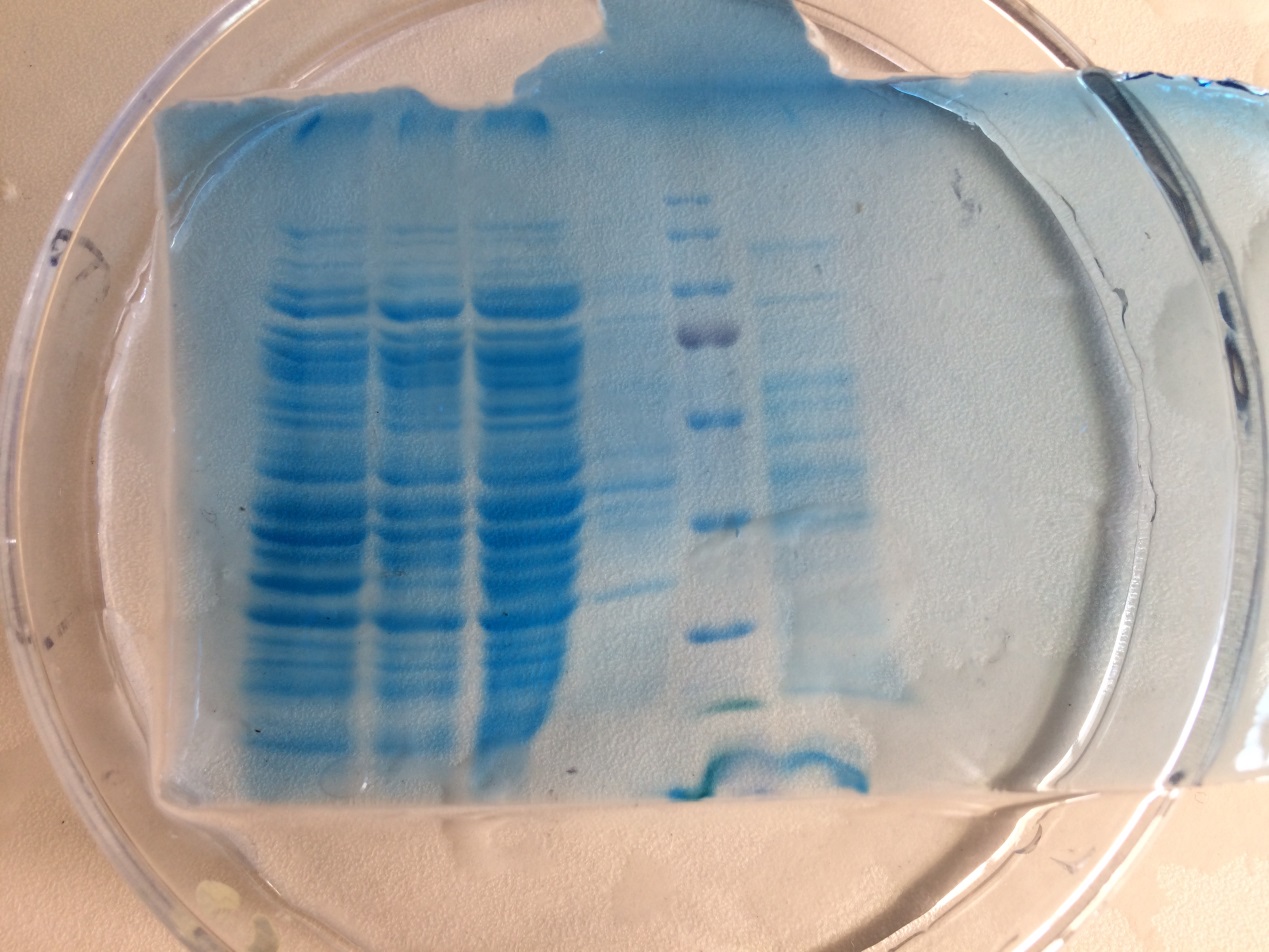

Supplement: Supplementary file 1 — Supplementary Information files [file 41598_2019_43172_MOESM1_ESM.docx]
